# The effect of Benson relaxation technique on the severity of symptoms and quality of life in children with irritable bowel syndrome (IBS): a quasi-experimental study

**DOI:** 10.1186/s12876-022-02631-0

**Published:** 2022-12-29

**Authors:** Saba Ebrahimloee, Anahita Masoumpoor, Malihe Nasiri, Mohadese Babaie, Amirparsa Vanaki Farahani, Sepideh Yousefiasl, Azam Shirinabadi Farahani

**Affiliations:** 1 Department of Pediatric Nursing, School of Nursing and Midwifery, Shahid Beheshti University of Medical Sciences, Tehran, Iran; 2 Student Research Committee, Department of Pediatric & Neonatal Intensive Care Nursing, School of Nursing & Midwifery, Shahid Beheshti University of Medical Sciences, Tehran, Iran; 3 Medical Student, School of Medicine, Shahed University, Tehran, Iran; 4 Nursing & Midwifery School, Shahid Beheshti University of Medical Sciences, Tehran, Iran; 5 Department of Pediatric & Neonatal Intensive Care Nursing, School of Nursing and Midwifery, Shahid Beheshti University of Medical Sciences, Tehran, Iran; 6 Department of Basic Science, Shahid Beheshti University of Medical Sciences, Tehran, Iran

**Keywords:** Irritable bowel syndrome (IBS), Symptom, Severity, Quality of life, Benson relaxation technique

## Abstract

**Aim:**

This study aimed to determine the effect of the Benson relaxation technique on the severity of symptoms and quality of life in children with irritable bowel syndrome (IBS).

**Design:**

This quasi-experimental study was carried out on children with irritable bowel syndrome in Iran.

**Method:**

Sixty children were randomly divided into control and experimental groups. The Benson relaxation technique was implemented for three weeks for experimental group, while the control group only received the typical medical therapy with no special intervention. The questionnaire of Irritable Bowel Syndrome-quality of life-34 (IBS-QOL-34), and Bowel Symptoms Severity and Frequency Scale (BSS-FS) were used for data gathering before and three weeks after the intervention. Data were analyzed using statistics, appropriate parametric and non-parametric tests.

**Results:**

According to the results, the experimental group had lower mean scores of qualities of life before the intervention (*p* < 0.05). The mean score of symptom severity in children with IBS was 13.88 in the experimental group, which changed to 9.83 in the post-test, indicating a significant difference (*p* < 0.000). The pre-test and post-test mean scores for quality of life in this group were 118.94 and 102.77, respectively, indicating a significant difference (*P* < 0.001).

**Conclusion:**

The Benson relaxation technique can be a non-pharmacological solution to reduce the severity of symptoms and improve the quality of life of children with IBS.

**Implication to practice:**

This technique is supposed to contribute as a further intervention in clinical contexts.

## Background

Irritable bowel syndrome (IBS) is a functional disorder of the gastrointestinal tract, diagnosed based on symptoms such as chronic abdominal pain and changes in defecation in the absence of any organic cause [1]. This disease is the most common gastrointestinal disorder and the cause of 12% of referrals to gastroenterologists [2]. In Iran, the overall prevalence of IBS is between 1.1 and 25% most of which is in women. There are no detailed studies or statistics on the prevalence of IBS in children, but the prevalence of this disease was estimated to be 4.2%, 12.6%, 16.4%, and 10.6% in students of Tehran, students of Gilan Universit of Medical Sciences, shiraz university, and Gorgan University, respectively [3].

This syndrome can increase health care costs directly and indirectly [4]. Therefore, health professionals have recently considered the health-related quality of life in this disease, given its increasing incidence, lack of definitive treatment, and the significant increase in health care costs. Evidence shows that patients with this syndrome have a significantly low quality of life [5]. Accordingly, these patients experience 3 to 4 times more absenteeism from work and education than healthy people. The disease also affects negatively social interactions, lifestyle, emotional relationships, leisure and travel, nutrition, and sleep of patients [6, 7].

Many patients with IBS are concerned about incontinence in the presence of others, which leads to their isolation. As a result, they become more and more lonely and experience low self-esteem, depression, and severity of symptoms, which can decrease their quality of life subsequently. Thus, measurement of health-related quality of life in IBS has been of great importance in the treatment of these patients, and improvement of their quality of life has been the main goal of therapeutic interventions [5].

There is no definitive medicine for this disease, and interventions only focus on alleviation of symptoms. However, clinical trials of psychological therapies, particularly cognitive behavioral therapy, have shown improvements. Randomized controlled trials have provided strong empirical evidence in support of the effectiveness of cognitive interventions as an adjunct therapy to routine therapy. Cognitive-behavioral therapy (CBT) and relaxation are two examples of these intervention [6, 8].

People can consciously change their physical, emotional, and stress-related behaviors through relaxation [9, 10]. There are several ways for relaxation [11], but the method proposed by Herbert Benson in 1970 is more desirable because it is more convenient to learn and teach. The Benson relaxation technique is based on attenuating sympathetic activity through an increase in the parasympathetic nervous system activity. Relaxation relieves emotional tension, alleviates pain-related muscle tension, improves sleep, and restores the individual sense of health [9]. Different studies have shown that the Benson relaxation technique decreases the severity of symptoms and anxiety in patients [12].

Children with irritable bowel syndrome (IBS) form a vulnerable group, and their quality of life is severely affected by the symptoms of this disease. So far, no research has been conducted to examine the effect of Benson relaxation on the symptoms and quality of life of these children. Therefore, the present study aimed to investigate the effect of the Benson relaxation technique on the severity of symptoms and quality of life in children with irritable bowel syndrome.

## Methods

### Trial design

This parallel quasi-experimental study was conducted from October 2019 to October 2020 in Tehran.

### Participants

Inclusion criteria were children aged 10–15 years, with irritable bowel syndrome who visited the gastroenterology clinic of the hospital or were hospitalized in the gastroenterology department of the hospital, Children whose severe pain caused by irritable bowel syndrome has led to hospitalization, ability to understand and speak Persian, no history of psychological disorders and hospitalization in psychiatric wards, no history of cancer Colon was in the family and had no other intestinal diseases. Children who were transferred to another hospital during the study or did not follow the planned intervention for more than 2 sessions were excluded from the study.

### Setting

Study setting was pediatric hospital, Tehran, Iran. Pediatric hospital is a subspecialty referral pediatric care center with different wards and units. The present study was conducted in one gastrointestinal wards of the hospital with 34 beds and gastrointestinal clinic.

### Sample

The study population included children aged 10–15 years with irritable bowel syndrome, referring to the clinic and gastrointestinal ward of Mofid Hospital affiliated to Shahid Beheshti University of Medical Sciences.

## Sample size calculation

According to the sample size formula $$\left( {n \ge {\raise0.7ex\hbox{${z_{1 - \alpha /2}^{2} \sigma^{2} }$} \!\mathord{\left/ {\vphantom {{z_{1 - \alpha /2}^{2} \sigma^{2} } {d^{2} }}}\right.\kern-0pt} \!\lower0.7ex\hbox{${d^{2} }$}}} \right)$$, the test power of 90% and the confidence coefficient of 95% [13], 60 participants made up the research sample, including 30 in each group. The required permission was first obtained from Shahid Beheshti University of Medical Sciences, after which coordination was made with the responsible authorities of the hospital, and then sampling began. The first researcher selected the eligible children from the ward and gastrointestinal clinic by the convenience sampling method and based on the inclusion criteria. Then, the children were divided into an experimental and a control group based on their file number (assigning the even numbers to the control group).

### Intervention

Benson relaxation technique was performed twice a day for a three-week period for the experimental group. On the first day of sampling, the relaxation technique was taught in a separate room in the ward. Parents were provided with a relaxation training CD, along with a training booklet to use in the case of discharge from the ward and at home until the end of the intervention period. For Benson’s relaxation technique, the child was asked to let go of disturbing thoughts and choose a word (like God) that has always been a reminder of calm, and begin to take deep, regular breaths (inhale by nose and exhalation by mouth), and repeat the word sedative. Participants were asked to simultaneously relax their muscles from the tips of their toes to the top of the body so that all the muscles of the body were fully expanded and this procedure was performed for 20 min. During this procedure, the child carefully followed the steps with the help of an audio file. The audio file of the instruction was set for 20 min and there was no need for the nurse to tune the clock to determine the duration of the procedure. After the training sessions and ensuring that the participants learned, they had to do this exercise twice a day (morning and afternoon), at least six hours apart, for three weeks, each time for 20 min. Regarding children who were treated on an outpatient basis or were discharged after hospitalization, the researcher also telephoned the participants twice a week to troubleshoot, strengthen, support, and verify compliance with the procedure. In hospitalized children, the researcher went to the child's bedside at the mentioned times and checked the method of performing the procedure.

The control group did not receive any special interventions, except the routine care of the ward or the typical training of the clinic. Sampling and intervention in the ward and clinic were performed in the morning and evening shifts by the researcher (a pediatric master student who received training in this field and had an educational certificate). At the end of the training period, the research tools were again provided to the children, referring to the clinic to follow up the treatment with the coordination of the researcher.

### Outcome measure

Assessing the severity of symptoms of IBS and the quality of life in Children with IBS were the primary outcome of this study.

Three instruments were used for data collection.

1. *Demographic and Clinical Questionnaire* This questionnaire included information, such as age, gender, last school degree, duration of disease, number of visits to the doctor over the past year, number of days absent from school due to illness over the past year, and chronic disease.
2. *Questionnaire of Irritable bowel syndrome—quality of life—34 (IBS-QOL-34)* This questionnaire was designed in 1998 by Patrick and Drossman for children aged 10–15 years. It has 34 items and 8 subscales, including health, prevention of (daily) activities, body image, health worries, food avoidance, social reaction, sexuality, and social relationships. The questionnaire is on a 5-point Likert scale at a range of 1 to 5 with a maximum score of 170 and a minimum score of 34. Scores 34 to 110 indicate a moderate intestinal disorder, and scores higher than 110 show a severe intestinal disorder. The internal consistency of this questionnaire was 0.95, according to Cronbach's alpha calculation. Also, Haghayegh et al. (2012) reported the internal consistency coefficient in the dimensions of this questionnaire as 0.52–88 and the total scale alpha of 0.93 [14].
3. *Bowel Symptoms Severity and Frequency Scale (BSS-FS)* This scale is based on Rome's diagnostic criteria and has 10 items at a 5-point Likert scale (0–4). It examines the severity of symptoms in patients with irritable bowel syndrome. The total score is 40 with higher scores indicating greater severity of gastrointestinal symptoms. Solati et al. [15] implemented this scale on patients with irritable bowel syndrome in Isfahan. The overall reliability coefficient of this scale was 0.80 using Cronbach’s alpha method.

The qualitative content validity of the research tools was assessed using the opinions of 5 faculty members of the School of Nursing and Midwifery and 5 pediatric gastroenterologists. The opinions of 10 research samples determined face validity [16], and the calculation of Cronbach's alpha determined the internal consistency. According to the results, the value of Cronbach's alpha was 0.87 and 0.79 for the total instrument in IBS-QOL-34 and BSS-FS, respectively. These questionnaires were completed before the initiation of the study and 3 weeks after the intervention.

### Data collection

Consent was primarily obtained from the children's parents to conduct the research. The children and their legal guardians received a complete explanation of the research tools with an emphasis on the confidentiality of the questionnaire data. The phone numbers of the parents were obtained to make the necessary calls, report on the end of the intervention, and explain the method of completing the research tools. Besides, the parents were provided with the researcher's phone number to resolve any ambiguity in performing the intervention or completing the research tools. On the first day, the Demographic and Clinical Questionnaire, IBS-QOL-34 scale, and BSS-FS scale were distributed as the research tools. For the comfort and convenience of mothers and children, a maximum of 24 h was considered to complete the questionnaires in the case of hospitalized children; however, those referring to the clinic completed the questionnaires at the time of referral. Completion of questionnaires took about 20 min. A blind researcher for randomization scored the questionnaires. The participants were asked to contact the researchers if they had any questions and not to receive information from other sources.

### Statistical analysis

The research variables were described using frequency tables. Appropriate parametric and non-parametric tests such as paired t-test were used to examine the relationship between variables based on the type of the variable under study in SPSS 22. *P*-values of less than 0.05 were considered statistically significant.

## Results

**Fig. 1:**
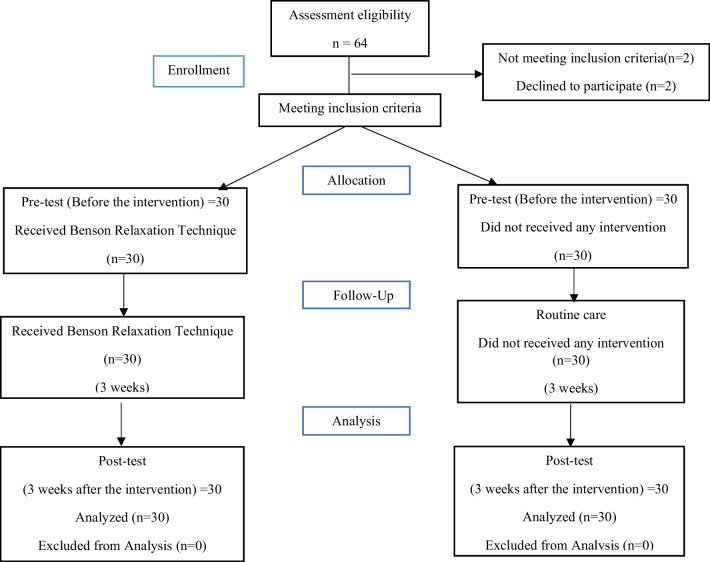
Diagram of CONSORT

A total number of 60 children with irritable bowel syndrome participated in this study. The participant attendance in the study is provided in Fig. 1.

The mean age of participants in the experimental and control groups was 12 and 12.26, respectively. The majority of the participants in the experimental group were male (53.3%), while the majority of the sample in the control group (60%) were female. The results of chi-square and independent t-test showed that there was no statistically significant difference between the participants in terms of demographic data. Table 1 shows the results of the demographic data.

**Table 1:** Demographic Data of participants in experimental and control groups

Variable	Experimental group (n = 30)	Control group (n = 30)	Sig
Gender			
Female	14 (46.7%)	18 (60%)	0.358^a^
Male	16 (53.3%)	12 (40%)	
School grade			
Elementary School	20 (66.7%)	22 (73.3%)	0.500^a^
Secondary School	10 (33.3%)	8 (26.7%)	
Number of visits			
< 10	26 (86.7%)	24 (80%)	0.500^a^
> 10	4 (13.3%)	6 (20%)	
Duration of disease			
< 1 year	4 (13.3%)	4 (13.3%)	0.884^a^
1–4 years	20 (66.7%)	22 (73.4%)	
> 4 years	6 (20%)	4 (13.3%)	
Days absent from school			
< 10	26 (86.7%)	26 (86.7%)	0.701^a^
10–20	4 (13.3%)	4 (13.3%)	
Age (mean)	121	2.26	0.619^b^

^a^chi-square test

^b^independent t-test

According to the results of Table 1, the average age of the samples was 12 years.

The mean pre-test score of symptom severity in the experimental group was 13.88, which changed to 9.83 in the post-test. Accordingly, there was a statistically significant difference in the mean pre-test and post-test scores of symptom severity in the experimental group (*p* < 0.000), and the mean post-test scores of symptom severity were lower than the mean of pre-test scores. The mean pretest score of symptom severity in the control group was 13.73, which changed to 13.53 in the post-test. Accordingly, there was no statistically significant difference in the mean pre-test and post-test scores of symptom severity in the control group (*p* = 0.271). Table 2 shows the information on the severity of symptoms and the results of paired t-test.

**Table 2:** Mean and SD of symptom severity before and after intervention in groups

Variable	Group	Condition	Mean	SD	Paired t-test		
					Statistic	Df	*P*-value
Symptom severity	Experimental (n = 30)	Pre-test	13.88	1.52	7.43	14	0.001
		Post-test	9.83	2.77			
	Control (n = 30)	Pre-test	13.73	1.33	1.14	14	0.271
		Post-test	13.53	1.40			

In general, the mean score of quality of life of the experimental group was 118.94 before and 102.77 after the intervention, indicating a significant difference (*P* < 0.0001). On the other hand, the mean score of total quality of life did not show significant differences in the pretest and posttest of the control group (*P* > 0.05). Table 3 shows the information on the quality of life and the results of paired t-test.

**Table 3:** Mean and SD of quality of life before and after intervention in groups

Variable	Group	Condition	Mean	SD	Paired t-test		
					Statistic	Df	*P*-value
Quality of life	Experimental (n = 30)	Pre-test	118.94	5.94	15.55	14	0.001
		Post-test	102.77	8.04			
	Control (n = 30)	Pre-test	120.20	5.49	1.33	14	0.204
		Post-test	119.73	5.68			

## Discussion

This study examined the effect of Benson relaxation technique on the severity of symptoms and quality of life in children with irritable bowel syndrome. For this purpose, the demographic variables were examined in both groups. It was observed that these variables were the same in both groups, which makes the results more generalizable. Also, the average scores of the severity of IBS symptoms and quality of life in children before the intervention did not differ between the two groups. This lack of difference increases the accuracy of the results and better investigation of the changes related to the Benson relaxation effect.

According to the results, the majority of participants had moderate symptoms of IBS, which is similar to the study of Masaeli et al. [17]. The results of the present study also showed that the severity of symptoms in children with irritable bowel syndrome was reduced by the Benson relaxation technique. This finding is approved by many studies, which indicate the efficacy of this intervention technique in reducing annoying symptoms in patients. Various studies have investigated the effectiveness of this technique with short-term and long-term interventions. In a study, six months of Benson relaxation therapy reduced the severity of symptoms in the experimental group [13]. van der Veek et al.’ study (2007) showed that short-term relaxation could reduce the severity of IBS symptoms [17]. The results of this study are also consistent with the results obtained by Keefer et al. [18]. Therefore, what is important is to perform high-quality relaxation interventions in these patients, which will help to improve annoying symptoms, reduce tension, and relaxes the patients.

Kuo et al. [12] emphasized that psychological intervention and relaxation could reduce the severity of symptoms in patients with IBS. The annoying symptoms of the disease contain muscle cramps, abdominal pain, bloating, diarrhea, and chronic constipation, which are considered the principal and influential factors in the quality of life and academic achievement of children with IBS. Accordingly, non-pharmacological methods to reduce the severity of symptoms have attracted attention in recent years [6]. Non-pharmacological manners are more acceptable for patients and their families due to easy implementation and low cost [19]. According to the results of the current and previous studies, the Benson relaxation technique can be used and taught to families and patients themselves to reduce the severity of symptoms in children with irritable bowel syndrome.

The present study’s results showed that the quality of life in children with irritable bowel syndrome was moderate, which is in line with the study of Tamannaifar and Akhavan-Hejazi [20]. None of the relevant studies has reported high quality of life for these patients. Given the low quality of life reported in both national and international studies, it seems necessary to provide solutions that can improve the quality of life of these patients. So far, different non-pharmacological methods have been examined, but no one has investigated the Benson relaxation technique and its effect on the quality of life of children with IBS. Therefore, the current study seemed completely necessary and practical. Regarding the effect of the Benson relaxation technique on quality of life, the results of the study showed that the Benson relaxation technique could improve the quality of life of children with irritable bowel syndrome. In the study of Asadi et al., although the participants were adults who underwent relaxation intervention, the obtained results confirm the findings of this study [1]. Relaxation techniques can seriously improve the quality of life in patients with irritable bowel syndrome [12, 18, 21].

### Practice implications

However, the use of Benson relaxation in child care is not common and most health care providers have limited information about it. Benson relaxation is an effective, simple, and transferable therapy that can be taught and used in many Places.

The findings of the present study can be used by nurses and other healthcare providers for children with IBS.

## Conclusion

This study showed that the Benson relaxation technique could reduce the severity of symptoms and improve the quality of life in children with irritable bowel syndrome. Thus, this technique can be taught to families with children suffering from irritable bowel syndrome at a macro level to provide comfort for the patients and their families. On the other hand, these patients can progress in the community when their quality of life improves. Given that this study was performed in one ward and one pediatric hospital, there are limitations in the generalization of the findings. In this research, we did not examine the differences in outcomes between those participants who were inpatient vs outpatient or those who were inpatient and released. Because most of the children were hospitalized for a while and because the research time was long (three-week period), they continued the procedure at home. But it is suggested that the difference between these two groups of children should be investigated in future studies.

### Strengths and weaknesses

Although, this hospital is a referral center for children all over the country, but there is a geographical, social and ethnic diversity in Iran. So, study sample might have not been representative of all children in Iran and in the world. Hence, findings may have limited generalizability. Further studies on larger samples of children are recommended.

## Data Availability

The datasets analyzed du The datasets generated and/or analyzed during ring the current study are not publicly available due [Because the information is about children who participated in the study and is therefore considered confidential] but are available from the corresponding author on reasonable request.

## Abbreviations

IBS: Irritable bowel syndrome
IBS-QOL-34: Irritable bowel syndrome-quality of life-34
BSS-FS: Bowel symptoms severity and frequency Scale
QOL: Quality of life
